# Sheep and farm level factors associated with footrot: a longitudinal repeated cross-sectional study of sheep on six farms in the UK

**DOI:** 10.1136/vr.104553

**Published:** 2018-01-23

**Authors:** Joseph William Angell, Dai H Grove-White, Jennifer S Duncan

**Affiliations:** 1 Department of Epidemiology and Population Health, Institute of Infection and Global Health, The University of Liverpool, Neston, UK; 2 Wern Veterinary Surgeons, Unit 11, Lon Parcwr Industrial Estate, Ruthin, UK; 3 School of Veterinary Science, University of Liverpool, Neston, UK

**Keywords:** footrot, lameness, sheep, epidemiology, risk factors

## Abstract

Footrot is an ovine foot disease of infectious origin and a cause of serious welfare and economic compromise in affected animals and flocks. The development of footrot in sheep is associated with the infectious agent *Dichelobacter nodosus*, which may invade as a primary pathogen, but the risk of disease is increased following damage to the interdigital skin of the foot. In this study, we used data from six farms in North Wales collected between June 2012 and October 2013 to model the dynamic changes of footrot prevalence over time and investigate the association of footrot with multiple farm, management, environmental and sheep factors. Footrot prevalence varied widely within and between farms and overall varied with season with an increase in prevalence shown in late summer and again in the spring. In addition, sheep were more likely to have footrot when the flock size was larger, when grazing poached pasture or when grazing a longer sward, and yearling sheep were less likely to have footrot when compared with lambs and adult sheep. These data may be helpful for advising farmers of likely environmental events, risk groups and management practices that may increase the probability of sheep developing footrot.

## Introduction

Footrot is an ovine foot disease of infectious origin and a cause of serious welfare and economic compromise in affected animals and flocks. It has long been established in the sheep-producing areas of the world and much has been done to address it.^1 2^

The development of footrot in sheep is associated with the infectious agent *Dichelobacter nodosus*, which may invade as a primary pathogen, but the risk of disease is increased following damage to the interdigital skin of the foot. Several workers have demonstrated how the prolonged presence of moisture could act as the traumatising agent presumably by macerating the interdigital skin,^3 4^ and large-scale eradication programmes have used prolonged dry periods of weather in order to assist in eradication.^5 6^ The UK has a temperate damp climate and the extrapolation of eradication strategies from some parts of the world are generally considered difficult to implement in the UK due to the absence of a prolonged and predictable dry period. Eradication in more temperate climates, for example, Norway and Switzerland^7^ has been successful; however, key differences which are likely to have aided success include fewer affected and more isolated flocks compared with the UK, government as well as industry investment together with a much closer engagement between vets and farmers. Therefore, due to a complex variety of factors including the climatic conditions, the endemic nature of the disease, the close proximity and geographical fragmentation of sheep farms, the hands-off approach of the UK government to endemic disease control, together with the perceived cost of eradication for individual farms, eradication in the UK is seldom practiced. Instead,  UK farmers are encouraged to maintain footrot to as low a prevalence as possible, for example, less than 2 per cent.^8^

Recent evidence from England implies that prevalence has reduced in recent years—possibly as a result of industry action in response to extensive knowledge exchange programmes based on practical intervention strategies.^9^ It was recently suggested that while national prevalence figures are widely used to describe levels of disease on farms and to provide targets for disease control,^8^ the use of point prevalence in this way is potentially unreliable and grossly oversimplifies what may be a dynamic situation, in that the prevalence of foot disease, or lameness, can vary widely over a year.^10^ Despite over a century of research into footrot, there is a paucity of robust observational evidence as to the expected dynamic changes in disease on farms in temperate climates—including the UK. This makes it difficult to credibly predict changes to the risk of an outbreak of disease on farms, and also leads to difficulties in interpreting point prevalence estimates for individual farms. Should such information exist, farmers and advisors could be alert as to critical high-risk periods or events in order to focus their resources in terms of control, and point prevalence estimates could be interpreted in light of expected fluctuations within a specified time period.

The data in this study were collected as part of an intensive observational study carried out on six UK farms to investigate risk factors associated with contagious ovine digital dermatitis (CODD) and represent the largest such study of its type.^10^ As such, these data allow further examination of the dynamic changes of footrot prevalence on these farms and the association with multiple farm, management, environmental and sheep factors allowing investigation of the changes in prevalence and risk of disease. Therefore, the aim of this study was to use this dataset to investigate these associations.

### Materials and methods

#### Study design and study population

The study protocol was approved by The University of Liverpool ethics committee (VREC 13) on 24 August 2011.

This study used a dataset collected as a result of a prospective, repeated cross-sectional study of six sheep farms in North Wales. The study design and data collection are described in detail by Angell and others.^10^ Briefly, six farms in North Wales were visited approximately every two months over a 12-month period (June 2012–October 2013). At each visit farm, group and sheep level data were recorded. All sheep on the farm were visually inspected in groups of approximately 10 sheep in handling pens. All sheep on the farm were lameness scored in the pen using a four-point ordinal locomotion scoring system.^11^ All lame sheep (score 1–3) were selected for further detailed examination, together with an equal or greater number of non-lame (score 0) control sheep, randomly selected from the same pen. Each selected sheep was examined in detail and covariate data recorded (Table 1). All the data collection, observations and examinations were made by the same person (JA) in order to reduce the risk of differential misclassification by different observers.

**TABLE 1:** Description of variables collected at sampling visits for initial inclusion in statistical analyses

Variable	Description and coding of variable
**Farm and environment**	
Farm id	Numerical identification numbers 1–6
Flock size at visit (n sheep)	1=210–400 2=401–800 3=801–1200 4=1201–1821
Land type	1=Hill 2=Upland/lowland 3=Lowland
Field stocking density	The number of sheep per hectare in each field at sampling 1=2.2–12.3 2=12.4–29.2 3=29.3–92.6
Pasture moisture	1=Dry 2=Damp and well drained 3=Wet 4=Boggy
Pasture quality	1=Lush 2=Average 3=Rough
Sward height (cm)	The mean compressed sward height in each field at sampling 1=1.3–5.0 2=5.1–10.0 3=>10.0
Sward cover	1=Good coverage 2=Patches 3=Heavily poached
**Sheep variables**	
Age	1=Lamb 2=Yearling 3=Adult
Body condition score	1=Very thin 2=Lean 3=Average 4=Fat 5=Obese
Perineal cleanliness	0=Clean 1=Mild staining 2=Dirty 3=Large dags
	0=Clean 1=Some dirt present
**Foot lesions**	
Footrot	0=No footrot present 1=Footrot present
Interdigital dermatitis (scald)	0=No interdigital dermatitis present 1=Interdigital dermatitis present
Active contagious ovine digital dermatitis (CODD)	0=No active CODD lesion present 1=Active CODD lesion present
White line lesion	0=No white line lesion present 1=White line lesion present
Overgrown	0=Overgrowth of hoof horn present 1=No overgrowth of hoof horn present

The study was designed to examine the prevalence of CODD on affected farms and farm, management, environmental and sheep factors associated with the presence of CODD lesions in sheep. However, due to the nature of the study these data could also be examined with respect to the clinical outcome footrot, which is the focus of this report.

Foot lesions were classified on the basis of their clinical appearance as CODD (together with grade) as per Angell and others,^12^ footrot as per the description by Egerton and Roberts^13^ and also used by Foddai and others.^14^ In some cases, feet could be considered to have features of both footrot and CODD, or interdigital dermatitis (scald) and CODD. In these cases, the combination was recorded. White line lesions^15^ and overgrown feet were also investigated as associated risk factors for footrot.

The covariate data considered in this study included flock size at visit, land type, field stocking density (sheep/hectare), pasture moisture, pasture quality, mean compressed sward height (cm), field sward cover, sheep identification number and locomotion score.^11^ Detailed methodologies for assessing the pasture are outlined by Angell and others^10^; however in brief, pasture moisture was assessed as: (1) ‘dry’, (2) ‘damp and well drained’, (3) ‘wet’, (4) ‘boggy’; pasture quality was assessed visually as (1) ‘lush’, (2) ‘average’, (3) rough’; pasture coverage was assessed visually as (1) ‘good coverage’, (2) ‘patches’—incomplete sward cover, (3) ‘heavily poached’ more than 50 per cent of the sward was absent. The mean compressed sward height was determined using a plate meter (Filips Manual Folding Plate Meter; Jenquip, Fielding, New Zealand),^16^ with the observer walking in a zigzag pattern taking recordings every 10 paces. Between 18 and 445 readings were taken per field (depending on size) to obtain the mean compressed sward height. Body condition score,^17^ age estimated from the number of incisor teeth present^18^ and breed were recorded, and cleanliness of the tail and perineal wool was recorded using an ordinal scoring system (0) ‘clean’, (1) ‘mild staining’, (2) ‘dirty’, (3) ‘large dags’ (hardened accumulations of faecal debris adhering to the wool) (table 1).

### Data analysis

All analyses were conducted using STATA V.14. For clarity statistical significance was set at P≤0.05; however, we considered that a less prescriptive approach to statistical significance may be more meaningful. Consequently, P values close to but yet >0.05 were considered weak evidence of an association and worthy of discussion.

### Prevalence and descriptive statistics

The point prevalence of footrot was calculated as the number of sheep affected as a proportion of the flock at the time of visit. Due to the sampling strategy, the true flock prevalence was estimated using probability weights as defined previously by Angell and others^10^:

$$P=\frac{\left(N_{case}\;+\;N_{case}\right)}{N_{tot}}$$

P=estimated prevalence;

N_case_=number of recorded cases in examined sheep—lame and not lame;

N_est_=estimated number of cases in unsampled flock—not lame;

N_tot_=total number of sheep in the flock.

The estimated number of cases in the unsampled flock (N_est_) was calculated from the sample data using the formula:

$$N_{est}\;=\left[\frac{NL_{case}}{NL_{tot}}\right]\;\times \;N_{uns}$$

NL_case_=the number of sheep with footrot that were not lame at examination;

NL_tot_=the total number of examined sheep that were not lame;

N_uns_=N_tot_ minus the total number of sheep sampled.

### Modelling

Logistic regression was employed to investigate associations between the binary primary outcome variable—the presence of footrot at sheep level—and potential farm, environmental, sheep and foot explanatory variables. The variable ‘flock size at visit’ was subdivided into four groups, and the variable ‘sward height’ was converted from a continuous variable into three categories 1.3–5.0 cm, 5.1–10.0 cm and more than 10.0 cm in order to allow more meaningful examination of the effect of these variables.

A multivariable logistic regression model was fitted using a backward elimination strategy, whereby a full model was fitted and then each variable removed in turn. Variables were retained if their removal led to a reduction in model fit. Interactions in the final model were considered for inclusion if considered plausible and retained if they improved model fit. The presence of CODD was excluded from these models due to the uncertainty of whether CODD is on the causal pathway between some of the explanatory variables and footrot or whether footrot is on the causal pathway for CODD. The final model was presented using all available observations for the variables included, excluding those with missing values.

Clustering of sheep within farm and in time due to the date of the visit were accounted for by fitting the model with farm and visit as random effects. The study design, whereby number of diseased sheep in the unsampled flock, estimated as described earlier, was accounted for using the SVY suite of commands in STATA V.14 and stratifying by lameness status (binary variable). Postestimation, a multivariable Wald test was carried out for those variables with more than one category to assess the overall strength of the association for the variable as a whole. To assess the overall model fit, the rank correlation of the presence of footrot at sheep level with the predicted probabilities was estimated by calculating Somers’ D, which may be equated to the area under the receiver operating characteristic (ROC) curve.^19 20^ Postestimation, the intraclass correlation coefficients (ICC) for the variables ‘farm id’ and ‘visit number’, fitted as random effects, were calculated from the variances using the following formulae:

Farm ID ICC=(variance farm)/((variance visit number) + (variance farm) + (π^2^)/3).

Visit number ICC=(variance visit number +variance farm)/((variance visit number) + (variance farm) + (π^2^)/3).

The final model was then used to assess the associations between the presence of footrot at sheep level and the included covariates adjusted for each other.

### Time

Seasonal changes in the prevalence of footrot were modelled by generating four time covariates (X_1_ X_2_ X_3_ X_4_) as follows: X_1_=cos(2πt/52), X_2_=sine(2πt/52), X_3_=cos(4πt/52) and X_4_=sine(4πt/52), where t=week of sampling. All four covariates were forced into all the models as a composite (harmonic regression)^21^, thereby allowing adjustment of all covariate estimates for the time of visit. The seasonal changes in prevalence per se were then described graphically by obtaining the logit prediction of a sheep having footrot estimated from a regression model, using the time covariates as the sole explanatory variables with the addition of probability weights to account for the non-random sampling strategy, and then calculating the inverse logit, that is, the prevalence. CIs were not presented due to there being only one farm visited on each day.

## Results

Six commercial sheep farms located in North Wales were sampled between 14 June 2012 and 3 October 2013. In total, 22 724 sheep were presented for inspection of which 6515 sheep were examined giving a sampling proportion of 28.7 per cent (95 per cent CI 28.1 per cent to 29.3 per cent).

### Prevalence and descriptive statistics for footrot

**FIG 1:**
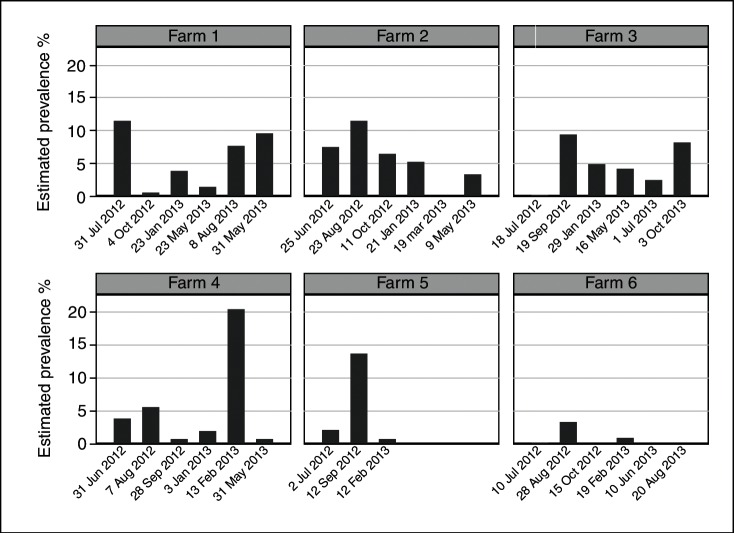
Estimated prevalence of footrot by farm determined at each visit. For farm 2 visit 5, and farm 6 visits 3 and 6, some of the data were missing preventing accurate prevalence estimates being calculated for these visits. For farm 5 visits 4–6, all data were missing from these visits due to the termination of the study on that farm.

Of the 6515 sheep examined, 526 had footrot (8.1 per cent (95 per cent CI 7.4 per cent to 8.8 per cent)). Of the 26 060 feet examined, there were 727 feet with footrot (2.8 per cent (95 per cent CI 2.6 per cent to 3.0 per cent)). As described previously, the estimated on farm prevalence of footrot was 5.0 per cent (95 per cent CI 3.2 per cent to 6.8 per cent)^10^ and the estimated prevalence of footrot varied by farm and visit (Fig 1).

For sheep over one year of age, 326 had footrot, and of these 46 had footrot on two separate occasions (14.1 per cent (95 per cent CI 10.7 per cent to 18.4 per cent)) and 12 had footrot on three occasions (3.7 per cent (95 per cent CI 2.1 per cent to 6.4 per cent)); 22 sheep (6.7 per cent (95 per cent CI 4.3 per cent to 10.0 per cent)) had footrot *in the same foot* on two separate occasions, and two individuals (0.6 per cent (95 per cent CI 0.1 per cent to 2.2 per cent)) had footrot *in the same foot* on three separate occasions.

### Multivariable analysis

The final multivariable logistic regression model for the presence of footrot at sheep level (n=5435 sheep) showed strong positive associations with larger flock size at the visit, poached ground and increased sward height (cm) (Table 2). A strong negative association was seen with yearling sheep compared with lambs and adult ewes (P<0.001), and a weak negative association seen with the presence of overgrown feet (P=0.05). Postestimation, Somers’ D was calculated as 0.76 (95 per cent CI 0.74 to 0.78), which when equated to the area under the ROC curve indicated that the model was a good fit of the data. The ICC for farm was calculated to be 0.04 and for visit number 0.08.

**TABLE 2:** Two-level multivariable logistic regression model including covariates associated with probability of diagnosing footrot in a sheep. In this model n=5435 sheep

Variable	OR	95 per cent CI	P value
**Farm and environment**			
Flock size at visit (210–400 as baseline)			
401–800	**3.8**	**2.1 to 6.9**	**<0.001**
801–1200	5.5	0.8 to 36.1	0.07
1201–1821	**5.6**	**1.2 to 24.9**	**0.03**
			0.006^*^
Stocking density (sheep/hectare) (2.2–12.3 as baseline)			
12.4–29.2	**1.9**	**1.1 to 3.5**	**0.04**
29.3–92.6	1.6	0.8 to 3.1	0.1
			0.1^*^
Pasture coverage (good coverage as baseline)			
Patches	1.8	0.7 to 4.8	0.2
Heavily poached	**2.8**	**1.1 to 6.9**	**0.03**
			0.007^*^
Sward height (cm) (1.3–5.0 cm as baseline)	**2.0**	**1.1 to 3.6**	**0.02**
**Sheep variables**			
Age (lamb as baseline)			
Yearling	**0.3**	**0.2 to 0.3**	**<0.001**
Adult	**0.6**	**0.4 to 0.8**	**0.005**
			<0.001^*^
**Foot lesions**			
Overgrown	**0.7**	**0.5 to 1.0**	**0.05**
**Time**			
X1	1.0	0.5 to 2.2	0.9
X2	0.5	0.3 to 1.0	0.04
X3	0.9	0.6 to 1.4	0.6
X4	1.2	0.6 to 2.5	0.5
**Random effects**	**Variance**	**se**	
Farm	0.1	0.2	
Visit number	0.2	0.2	

*P values refer to those as a result of a multivariable Wald test carried out postestimation.

### Time

**FIG 2:**
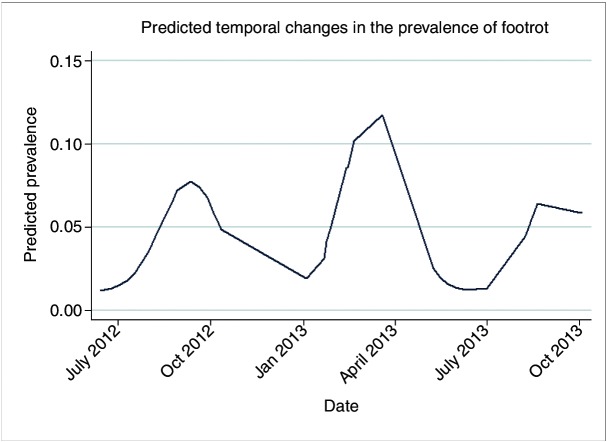
Predicted temporal changes in the prevalence of footrot.

The prevalence of sheep with a footrot lesion, across all six farms over time was predicted from a model containing the four time covariates (Fig 2). This showed significant variation in the prevalence of footrot over the six farms over time. In 2012, a significant increase in prevalence was observed in late summer/early autumn, and in 2013 a significant increase in prevalence was observed in the spring. There may also have been a further increase in prevalence in late summer/early autumn in 2013, before the termination of the study.

## Discussion

### Study limitations

These have been discussed previously by Angell and others^10^; however, in summary the non-random sampling strategy was employed to identify the largest number of sheep with CODD as per the original study question. This would inevitably lead to biased prevalence estimates, which were therefore addressed using probability weights to estimate prevalence. In addition, using this sampling approach, at each visit a proportion of sheep were resampled from previous visits together with a proportion that had not been sampled previously. This may have led to further bias being introduced due to some sheep appearing more than once in the dataset and observations relating to these individuals could clearly not be classed as independent. However, this lack of independence was addressed by fitting the multivariable model with farm and visit as random effects therefore adjusting the ORs to account for this clustering within farm and in time. It is also worth stressing that these data refer to six commercial farms in North Wales. While much of these data may hold true in principle for other farms—particularly within the UK, there are likely to be large variations between farms and geographical areas, dependent on altitude, soil type and local weather conditions. Therefore, while it may be interesting to consider specific examples from this dataset, the broad principles rather than exact numbers are likely to be more generalisable.

### Prevalence

Despite apparent reductions in England,^9^ footrot remains widespread and in this study there was wide variation between the six farms and within each farm over a year (Fig 1). For example, for farm 4 the estimated prevalence of footrot varied between 20.5 per cent at visit 5 and 0.5 per cent at visit 6. This reiterates the issues raised previously when considering the prevalence on a farm, in that a single estimate at just one point in time does not accurately describe disease levels on a farm and using a single measure to benchmark farms is likely to be inaccurate.

Some as yet unvalidated but widely publicized advice on controlling footrot advocates the culling of sheep that are repeatedly infected with footrot (‘repeat offenders’) as one of the mainstays of footrot control on affected farms.^22 23^ In this study, 58 sheep (17.8 per cent (95 per cent CI 14.0 per cent to 22.4 per cent)) affected with footrot were recorded as having a footrot lesion more than once. This represents a small number of sheep (between 4 and 22 per farm), thereby representing a potentially affordable intervention for many farms. If demonstrated to be efficacious for control then it is likely that such a policy will only result in a small economic cost to farmers. Furthermore, given the endemic nature of the disease in the UK, using the clinical appearance of lesions as the sole diagnostic tool is likely to be adequate for current culling strategies. More advanced diagnostic tools, for example, RT-PCR would be potentially useful should a concerted coordinated national eradication programme be instigated.

In addition, when considering the use of figures to benchmark farms against each another, the use of figures indicating the number of repeat offenders or number of sheep culled for footrot could be potentially misleading with farms adopting more or less rigorous approaches to footrot control reporting similar or counterintuitive results. For example, a farmer with a lax approach to footrot control and potentially higher incidence rates may cull a larger proportion of sheep with footrot, as may a farmer who is diligently recording sheep that develop footorot on two separate occasions.

### Factors associated with footrot

After adjusting for all the other factors in the model, sheep were more likely to have footrot if they were part of larger flocks, were grazing poached ground or were grazing a longer sward. To interpret what this might look like in a flock, the marginal mean and se were computed using the multivariable model presented (Table 2), postestimation, for adult sheep in a flock of between 401 and 800 ewes (average size for the UK (size 396–600)), grazing a sward of 5–10 cm in length. This resulted in a marginal mean probability of infection of 0.06 (se 0.02), that is, roughly 1 out of 17 sheep would have footrot.

In this study, sheep were more likely to have footrot when the flock size at the visit was larger. However, in a recent questionnaire study, larger flocks had a lower relative risk of footrot compared with smaller ones, although this may have been biased possibly by the small number of hill flocks included.^9^ For example, Grogono-Thomas and Johnston^24^ showed that lameness was less prevalent in large hill flocks and the inclusion of large hill flocks by Winter and others^9^ may therefore affect this result. In the UK, sheep flock size changes greatly over the year with the seasonal influx and then sale of lambs. As flock size increases, it is possible that it is harder to identify and manage individuals with disease promptly—as is recommended,^8 22 25 26^ particularly if the grazing is extensive and on difficult terrain.

It has long been shown that there is a causal association between maceration of the interdigital skin and the development of footrot—this process facilitating colonisation with *D nodosus*.^3^ This may be facilitated by other pathogens, for example, *Fusobacterium necrophorum*,^4^ moisture or possibly rough plants or rough ground abrading the interdigital skin.^3 27–29^ In this present study, there was a strong association with pasture coverage and sward height, although pasture moisture was not retained in the final model. Longer swards may hold more moisture for longer compared with shorter swards—acting like a sponge. This positive association with sward height may reflect that sheep standing in longer swards have their feet in a more extensive humid microclimate compared with those on shorter pastures (which may dry more rapidly), and while the pasture may not appear particularly wet, it may be sufficiently so at foot level to encourage skin maceration. Pastures with less grass cover and more exposed soil leading to poaching may damage and abrade the feet more, traumatising the skin and increasing the risk of bacterial colonisation.

As was found for CODD by Angell and others^10^, yearling sheep were much less likely to have footrot compared with lambs or adults, and this may reflect the selection of quality replacement sheep and the maintenance of these animals in a separate group, with different management, until lambing as adults. It may also reflect the fact that these sheep may be under less physiological stress in that their growth rate is much lower compared with a lamb and they are kept on many farms in a non-productive state, for example, not pregnant or lactating.

In the study by Angell and others^10^, there was a strong association between the presence of footrot and the presence of CODD. However, due to the cross-sectional nature of the study it was not possible to determine the temporal association between these two lesions, that is, does footrot precede CODD, CODD precede footrot or do both occur concurrently as ‘contagious ovine foot disease’. It is possible that all three scenarios are true and further work is necessary to unravel this. However, there is increasing evidence that *D nodosus* may precede CODD, for example, Duncan and others^30^ demonstrated that vaccination against *D nodosus* reduced the prevalence of CODD. For this reason, CODD was not included in the multivariable models and more work is needed to understand the interaction between these two diseases. With the current knowledge, we would suggest that farmers and veterinary surgeons develop holistic approaches to controlling contagious foot diseases as neglecting footrot while dealing with CODD, or neglecting CODD while dealing with footrot may lead to worse than expected results and frustration.

In this study, the ICC for both farm and visit number was small: 0.04 and 0.08, respectively. This implies that the effect of farm and visit number was relatively small compared with the other covariates in the model, suggesting that these other factors (eg, if they were part of larger flocks, were grazing poached ground or were grazing a longer sward) are more important in terms of a sheep having footrot.

### Seasonal changes in footrot

In this study, an increase in footrot prevalence was observed in late summer/early autumn in 2012. This is consistent with seasonal fluctuations observed by Clements and Stoye^31^ on one farm in England. There was also an increase in prevalence in the spring of 2013, which is consistent with an increase in infection pressure following housing for lambing, a practice which occurred on five of the six farms. This is consistent with the study by Whittington,^29^ who also showed the occurrence of transmission between infected and uninfected sheep via contaminated handling pens.

## Conclusions

These data may be helpful for advising farmers of likely environmental events (seasonal risk), risk groups (adults) and management practices (grazing poached pasture and on longer swards) that may increase the probability of sheep developing footrot.
